# Coupled electrophysiological recording and single cell transcriptome analyses revealed molecular mechanisms underlying neuronal maturation

**DOI:** 10.1007/s13238-016-0247-8

**Published:** 2016-02-16

**Authors:** Xiaoying Chen, Kunshan Zhang, Liqiang Zhou, Xinpei Gao, Junbang Wang, Yinan Yao, Fei He, Yuping Luo, Yongchun Yu, Siguang Li, Liming Cheng, Yi E. Sun

**Affiliations:** Stem Cell Translational Research Center, Tongji Hospital, Tongji University School of Medicine, Shanghai, 200065 China; Department of Psychiatry and Biobehavioral Sciences, David Geffen School of Medicine, University of California, Los Angeles, CA 90095 USA; Institute of Neurobiology, Institute of Brain Science and State Key Laboratory of Medical Neurobiology, Fudan University, Shanghai, 200032 China; Collaborative Innovation Center for Brain Science, Tongji University, Shanghai, 200092 China

**Keywords:** Patch-Seq, hESC/hiPSC-derived neuron, WGCNA, Biomarkers for neuronal maturation, Ubiquitination and mitochondrial function

## Abstract

The mammalian brain is heterogeneous, containing billions of neurons and trillions of synapses forming various neural circuitries, through which sense, movement, thought, and emotion arise. The cellular heterogeneity of the brain has made it difficult to study the molecular logic of neural circuitry wiring, pruning, activation, and plasticity, until recently, transcriptome analyses with single cell resolution makes decoding of gene regulatory networks underlying aforementioned circuitry properties possible. Here we report success in performing both electrophysiological and whole-genome transcriptome analyses on single human neurons in culture. Using Weighted Gene Coexpression Network Analyses (WGCNA), we identified gene clusters highly correlated with neuronal maturation judged by electrophysiological characteristics. A tight link between neuronal maturation and genes involved in ubiquitination and mitochondrial function was revealed. Moreover, we identified a list of candidate genes, which could potentially serve as biomarkers for neuronal maturation. Coupled electrophysiological recording and single cell transcriptome analysis will serve as powerful tools in the future to unveil molecular logics for neural circuitry functions.

**Electronic supplementary material:**

The online version of this article (doi:10.1007/s13238-016-0247-8) contains supplementary material, which is available to authorized users.

## INTRODUCTION

The mammalian brain is a fascinating organ, which breeds wisdom, empathy, and creativity, i.e., features unique to the human mind. The brain is also particularly complex, composed of billions of neurons and trillions of glia including both astrocytes and oligodendrocytes. Moreover, billions of neurons form trillions of synapses, which are critical points of communications between and amongst neurons. Through synaptic activities, neurons are integrated and organized in networks and circuitries, where senses, movement, thought, and emotions are generated. Interestingly, it is believed that when an individual is conducting a specific task, only a few neurons or specific neural circuitries are engaged in the activity, which means analysis using brain tissues, as a whole will not provide sufficient resolution for dissection of the molecular gene network underlying the particular brain function. While it is known that cells could be pretty much defined by the genes that they express, and that transcriptome analyses have been shown to be useful to delineate molecular features associated with cell type, cell age, and cell physiological or pathological states, transcriptome analyses of brain tissues had not been particularly useful due to the huge cellular heterogeneity of brain tissues and that cells of interest are often rare populations, such as neurons in particular circuitries involved in specific behaviors. Although there are some bioinformatics tools including WGCNA, which can be used to sort out cell-type specific gene expression signatures from transcriptome of the brain tissue (Miller et al., 2014; Kang et al., 2011; Mirnics 2008), a physical limitation is that transcription signals from rare cells in the tissue will simply not be captured when the whole tissue is subjected to RNA sequencing.

To get around these limitations, single neuron transcriptome analyses were developed, and many papers published on brain cell typing (Junker and van Oudenaarden 2014; Johnson et al., 2015; Pollen et al., 2015). However, most studies using cell sorting including the Fluidigm C1 apparatus completely lost the anatomical positional information of cells by taking them out of their natural habitat. Moreover, the dissociation process is a clear stress, if not devastating to the cells, particularly neurons, which could change the transcription program quite dramatically. It is therefore advantageous to perform transcriptome analyses on neurons that are in electrophysiologically functional state, so that molecular genetic programs underlying neuronal physiological properties could be revealed. In this study, we performed electrophysiological recordings on cultured human neurons differentiated from various sources including human ES cells, iPS cells, and embryonic neural stem cells. After recording, the same neurons were subsequently subjected to RNA sequencing. From single neuron transcriptome analyses, we identified gene programs associated with neuronal maturation judged by electrophysiological parameters. Moreover, by cross-referencing transcriptome data obtained from single human neurons in embryonic and adult human brains (Darmanis et al., 2015), we isolated 39 genes, which could be potentially used as neuronal maturation biomarkers. The methodology established here could also be used to couple single neuron electrophysiological and transcriptome analyses after *in vivo* or brain slices recording to reveal the molecular logic of neural circuitry activities.

## RESULTS

### *In vitro* differentiation and maturation of human neurons derived from hESCs and hiPSCs

**Figure 1:**
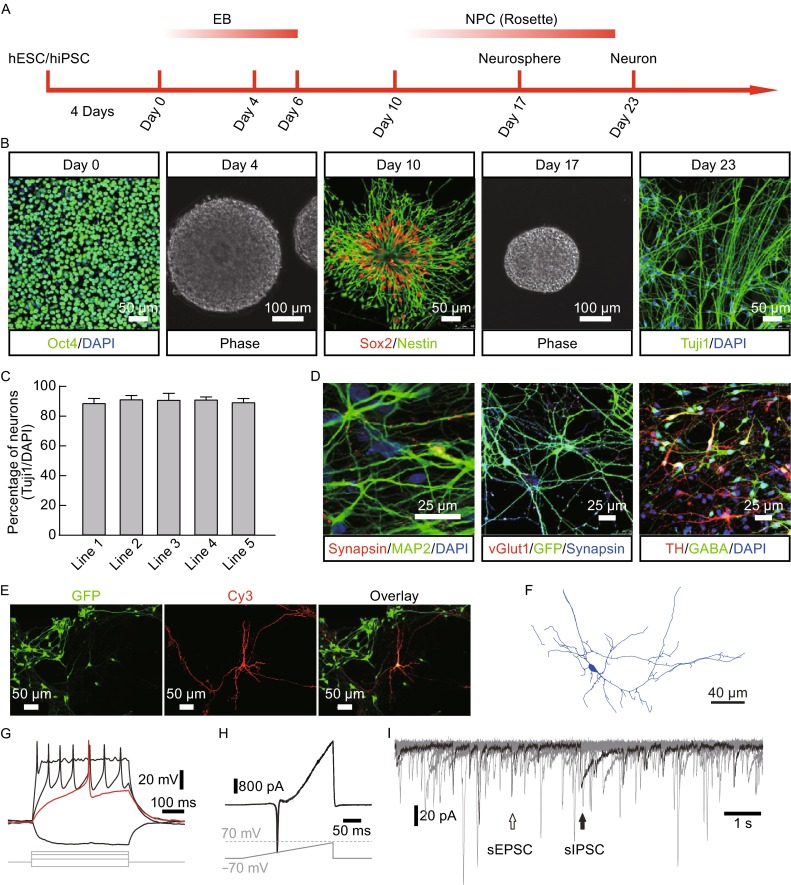
**Generation of functional neuron via differentiation of hESC/hiPSC/fetal tissue-derived NSCs**. (A) Diagram of the neuronal differentiation protocol (see experimental procedures for details). (B) Representative images showing morphological changes during neural differentiation. hESC/hiPSC are positive for pluripotent markers Oct4. Neural stem/progenitor cell (NSC/NPC) colonies, showing rosette structures, are positive for neural precursor markers Sox2 and Nestin, whereas neurons are positive for Tuj1 (Bar: 100 or 50 μm). (C) Percentage of Tuj1 positive cells showing similar pan neuronal differentiation potentials of different hESC/hiPSC lines. (D) Representative images of mature neurons, co-cultured with mouse astrocytes, showing expression of MAP2, Synapsin, vGlut1, TH, and GABA (Bar: 25 μm). (E) Representative images of a recorded neuron injected with neurobiotin and immunostained (Cy3), surrounded by other GFP expressing lentivirally infected neurons. Bar represents 50 μm. (F) A representative reconstructed image of neurons. Scale bar represents 40 μm. (G) Representative action potential responses of a hiPSC-derived neuron evoked by current injection (Bars: 100 ms and 20 mV). (H) Representative traces from a hiPSC-derived neuron showing inward sodium currents and outward potassium currents in response to a ramp protocol (from −70 mV to +70 mV). Three super imposed traces are shown (Bars: 50 ms and 800 pA). (I) Representative traces from a hiPSC-derived neuron showing spontaneous postsynaptic currents (sPSCs). Ten traces are superimposed. Black arrow, inhibitory sPSC; white arrow, excitatory sPSC (Bars: 1 s and 20 pA)

Ever since Thomson first established human embryonic stem (ES) cell cultures and Yamanaka developed human induced pluripotent stem cell (hiPSC) systems, human neurons could be readily obtained from *in vitro* differentiation and maturation (Wu et al., 2007; Zhang et al., 2013; Hu et al., 2010). Subsequently, human cell-based “disease-in-dish” models became popular approaches for attempting to study human neurological diseases (Mariani et al., 2015; Li et al., 2013; Ma et al., 2012). The step-wise neuronal differentiation protocols with all kinds of variations have been utilized by many laboratories to generate human neurons *in vitro* with high enrichment (Fig. 1A–C). Moreover, these neurons do mature in culture and form synaptic networks, which could be judged anatomically by presynaptic synapsin immunostaining puncta on postsynaptic MAP2-positive dendrites (Fig. 1D), or functionally by the presence of spontaneous excitatory and inhibitory postsynaptic currents (sEPSCs and sIPSCs) (Fig. 1I). As expected, we frequently detected vGlut1 positive glutamatergic excitatory neurons, GABA positive GABAergic inhibitory neurons, and TH positive catecholaminergic neurons (Fig. 1D). Using fluorescent dye injection, neuronal morphology could be precisely revealed and quantifiably measured (Fig. 1E and 1F). Some of these human neurons fire action potentials upon depolarization (Fig. 1G and 1H). However, a great degree of heterogeneity are clearly present in these neuronal cultures, regarding neurotransmitter-based neuronal subtypes, neuronal morphologies, and electrophysiological properties such as action potential firing frequencies, amplitude, and etc.

### Coupling of electrophysiological recording and transcriptome (Patch-seq) analyses on the same neurons

**Figure 2:**
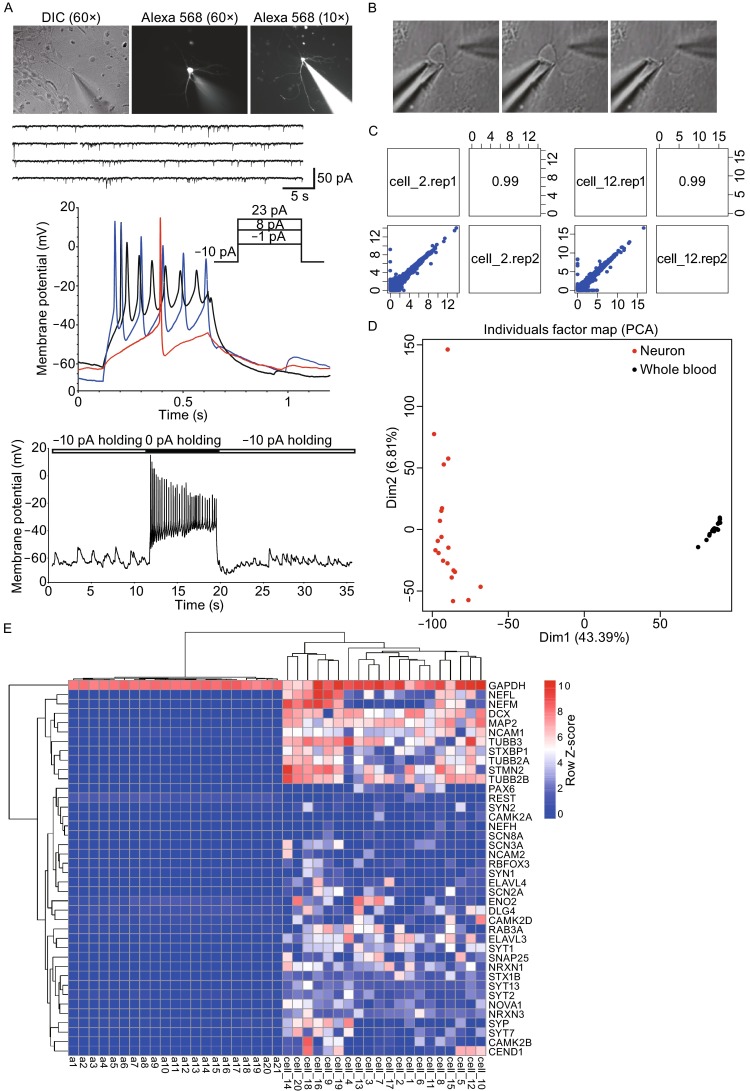

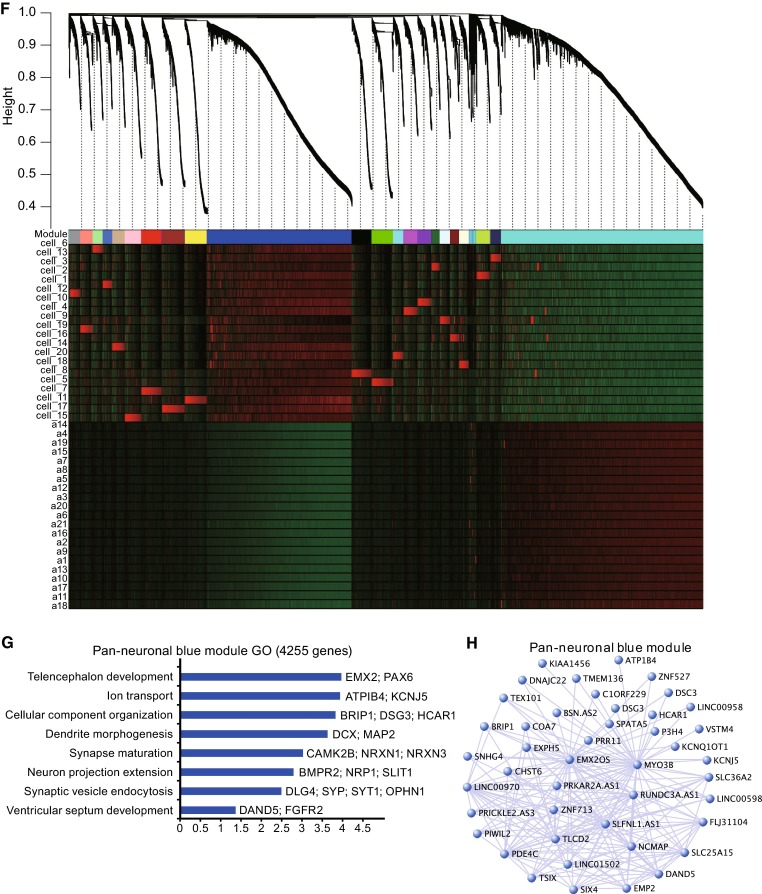
**Single cell transcriptome analyses reveal neuronal property of collected target cells**. (A) A representative patch-clamp recording of a H9 hESC-derived neuron. This neuron was filled with Alexa Fluor 568 hydrazide during whole-cell patch clamping to reveal the morphology (middle and right in top panel). Spontaneous postsynaptic currents were recorded through voltage-clamp mode (middle top panel). Short-term (0.5 s, middle top panel) and long-term (8 s, bottom panels) recordings of action potentials were performed with stepwise current injection through current-clamp mode. (B) Micromanipulator-assisted manual collection of single neurons showing moments of target cell entering the patch pipette, before (left), during (middle), and after (right) the entry. All soma and majority of dendritic parts were collected. (C) Technical replica of two single-cell transcriptome (Cell_2 and Cell_12) were subjected to two independent sequencings in two batches. High similarity between two replicates (Pearson correlation coefficient = 0.99) indicates minimum sequencing batch effect, hence the robustness and accuracy of our sequencing technology. (D) PCA of single-cell transcriptomes including neuron and peripheral blood samples suggested that neurons were distinct from peripheral blood cells. (E) Pan-neuronal genes were exclusively expressed in neurons but not blood samples. (F) Gene clustering of all 20 single cells and 21 blood samples (a1–a21) revealed a neuronal specific gene module (blue module). (G) GO analyses of the blue module (4255 genes). Length of bars indicates the significance (−log10 transferred *P*-value, Fisher’s exact test). Genes shown in right are well-known genes with corresponding functions. (H) Hub-gene network of the blue module

In order to delineate the molecular signatures underlying the heterogeneity of the electrophysiological properties of cultured human neurons mentioned above, we carried out “patch-seq” using our own proprietary method (Fig. 2). We measured 9 electrophysiological parameters, among which 6 were related to action potentials, i.e., firing rate, amplitude, halfwidth, threshold, Rin, and rise time (Fig. 2A). We also measured sodium current amplitude, as well as frequencies of sEPSCs and sIPSCs, which are indicative of neural network activities. After recording, neurons were individually extracted by the patch pipet (recording electrode) (Fig. 2B), and subjected to single neuron transcriptome analyses. We do frequently perform technical replications for sequencing to make sure that the sequencing quality is high with low noises, which could be judged by high Pearson Correlation Coefficient (~0.99) of log-transformed whole transcriptome between the replica (Fig. 2C). In this study we sequenced 20 single human neurons with various electrophysiological properties. When single neuronal transcriptome were compared with transcriptome of 21 human peripheral blood samples, we found that these two types of samples are clearly distinguished from each other by dimension 1 of the two-dimensional principle component analyses (PCA) (Fig. 2D). Expression of a list of well-acknowledged neuronal markers could clearly segregate blood samples from single neuron samples (Fig. 2E). Using WGCNA, we identified a gene module (blue, containing 4255 genes) that appeared to be neuronal cell specific (Fig. 2F). Gene Ontology (GO) analysis demonstrated that major GO terms associated with the blue module were indeed related to neurons including “synapse maturation”, “neuronal projection extension”, “dendrite morphogenesis”, “synaptic vesicle endocytosis”, and “telencephalon development” (Fig. 2G). Moreover, we also identified the hub-gene network of this neuronal-specific blue module (Fig. 2H). As expected, these hESC and iPSC-derived neurons tend to take on an anterior and dorsal trait as judged by gene expression, yet these cultures are by no means homogenous.

### Categorizing immature, maturing, and matured neurons based on electrophysiological properties

**Figure 3:**
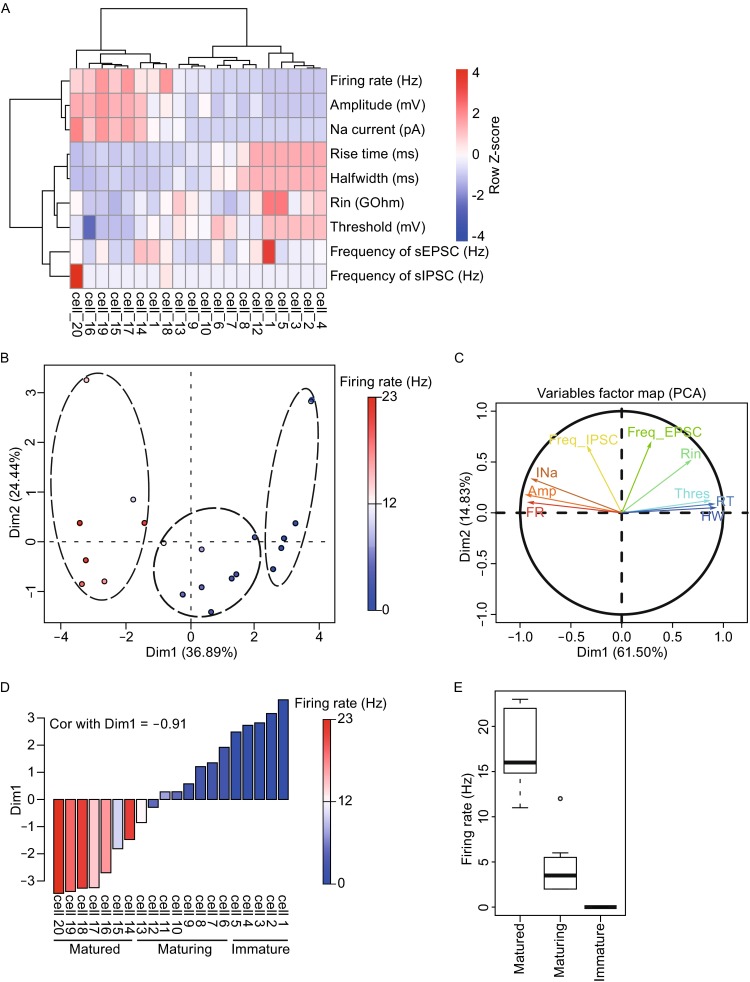
**Electrophysiological phenotypes of neurons revealed three groups of neurons based on their maturity**. (A) AP and sPSC electrophysiological parameters indicated different maturation stages of 20 single neurons. (B) PCA revealed dimension 1 as an indication of maturity and split 20 neurons into three groups. Colors of each circle correspond to different firing rate of the neurons, which is a highly regarded parameter for neuronal maturity. (C) Variable factor map revealed the correlation vector of each electrophysiological parameter with dimension 1 and dimension 2. Arrow tip denotes correlation coefficient of the respective parameter with each PC. Abbreviation, Thres. (Threshold); Freq_EPSC. (Frequency of sEPSC); RT. (Rise time); HW. (Halfwidth); Freq_IPSC. (Frequency of sIPSC); INa. (Na current); Amp. (Amplitude); FR. (Firing rate). (D) High absolute value of Pearson correlation coefficient (−0.91) between Dim1 and firing rate, indicating Dim1 is highly anti-correlated with neuron maturation. (E) Firing rate of three groups of neuron

The 9 parameters of electrophysiological properties of 20 human neurons were subjected to non-hierarchical clustering (Figs. 3A and S1, Table S1). Based on two-dimensional PCA, neurons could be divided into three groups along the first dimension (Dim1) (Fig. 3B). The variable factor map of the PCA is presented in Fig. 3C, from which it was clear that Dim1 mostly reflected 6 parameters describing different perspectives of action potentials. For example, Firing rate and Amplitude are negatively correlated with Dim1. On the contrary, Halfwidth, Rin, Rise Time, and Threshold are positively correlated with Dim1. This is expected because these 4 parameters are known to be negatively correlated with firing rate and amplitude (Fig. 3C). Sodium current, on the other hand, appeared to be positively correlated with firing rate and amplitude. Since firing rate is one of the best markers for neuronal electrophysiological maturation properties, the –0.911 anti-correlation value between Dim1 and firing rate suggested that either Dim1 or firing rate could be used to bin the 20 neurons into three groups, i.e., the immature group, the maturing group, and the matured group (Fig. 3D and 3E). It is worth noting that frequencies of sEPSCs and sIPSCs do not correlate well with Dim1 or maturation. This makes sense as sEPSCs and sIPSCs only reflect neural network activities imposed on the cell recorded, which does not necessarily mean whether the recorded neuron itself is mature or not, or whether that neuron could transmit the electric signal down to the axon or not (Fig. 3C). Given that Dim1 took all parameters into account, it should be more reliable and less prone to fluctuations as compared to any one parameter including the firing rate. Moreover, since Dim1 correlated with the firing rate very well, it is more sensible to use the Dim1 value to quantify neuronal maturation in order to dissect the underlying gene expression signatures.

### Gene programs positively and negatively correlated with neuronal maturation

**Figure 4:**
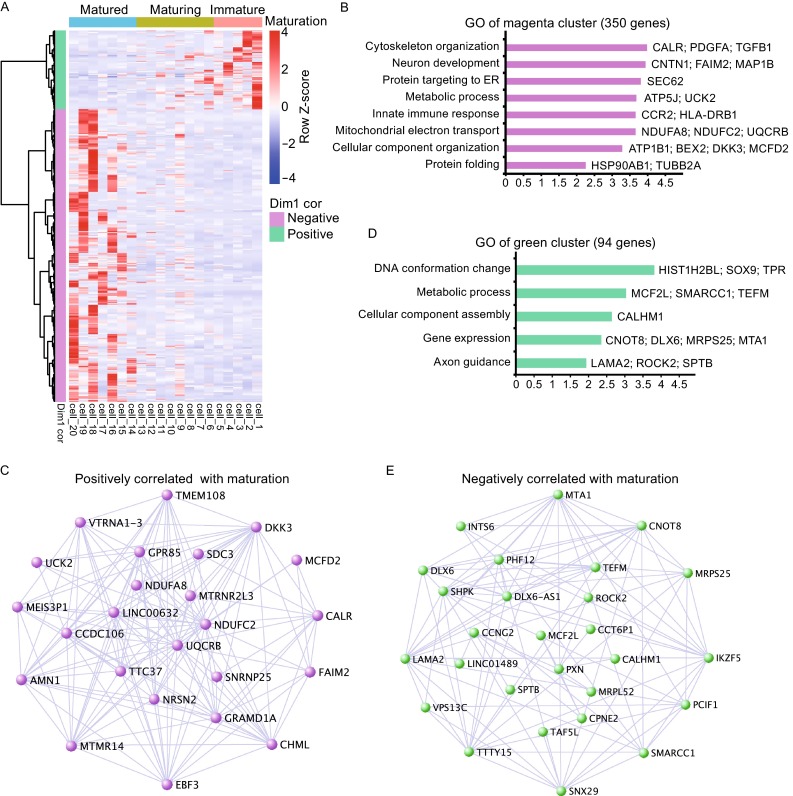
**Single cell transcriptome analysis revealed maturation-related genes**. (A) Expression of genes positively (green cluster) and negatively (magenta cluster) correlated to dimension 1 of electrophysiological parameters (Correlation > 0.4 and *P* < 0.05, Student correlation *P*-values) in 20 single neurons. (B) GO analyses of genes negatively correlated to dimension 1 (magenta cluster, 350 genes), enriched in mature neurons. Length of bars indicated the significance (−log10 transferred *P*-value, Fisher exact test). (C) Hub-gene network of the magenta gene cluster. (D) GO analyses of genes positively correlated with dimension 1 (green cluster, 94 genes), enriched in immature neurons. Length of bars indicated the significance (−log10 transferred *P*-value, Fisher exact test). (E) Hub-gene network of the green gene cluster

**Figure 5:**
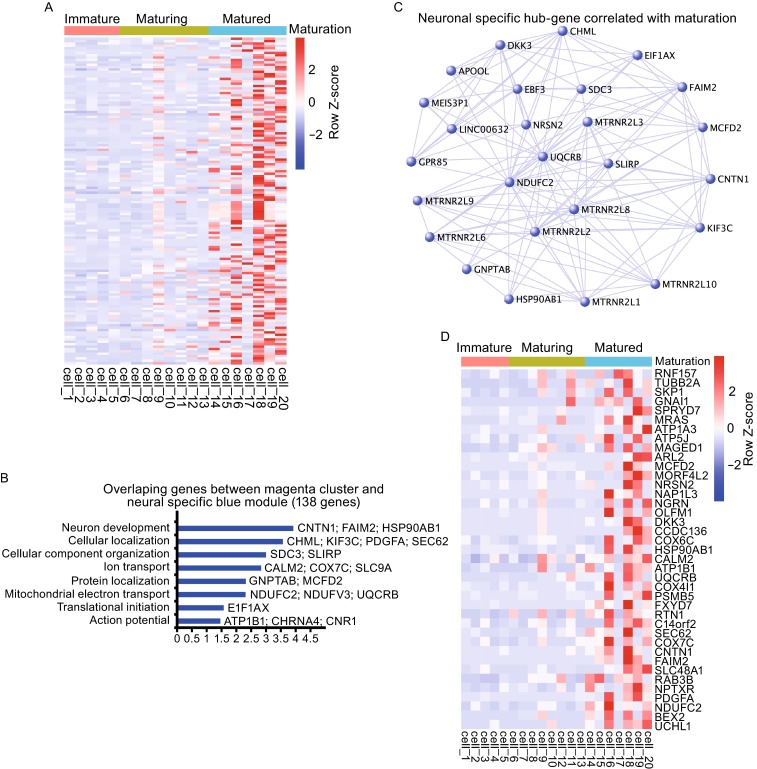
**Identification of putative generic neuronal maturation biomarkers**. (A) Expression of 138 genes in 20 single cells, which are shared between the magenta cluster and the blue module. (B) GO analyses of 138 genes. Length of bars indicated the significance (−log10 transferred *P*-value, Fisher exact test). (C) Hub-gene network of the 138 genes. (D) 39 putative generic neuronal maturation genes that were also reported in Darmanis et al., (2015)

From transcriptome of 20 single neuronal samples, we identified gene clusters that were negatively correlated with Dim1 of the electrophysiological parameters, which meant that these genes were enriched in matured neurons. We also identified genes that were positively correlated with Dim1, indicative of their affiliation to immature neurons (Fig. 4A). Clearly when neurons transit from an immature to a mature state, they will first shut off signature genes associated with immature neurons (the green cluster, 94 genes) and gradually turn on signature genes affiliated with mature neurons (the magenta cluster, 350 genes) (Fig. 4A). The immature green gene cluster contained genes involved in “DNA conformation changes”, “gene expression”, and “axon guidance”, which were activities associated with early neuronal differentiation. Genes including Sox9, Dlx6, MTA1, LAMA2, ROCK1, and ROCK2 are highly expressed in immature neurons. On the other hand, GO analyses of the magenta cluster revealed genes linked to “neuron development” such as CNTN1, FAIM2, which were also hub-genes in the magenta cluster. Moreover, ATP1B1, a gene encoding a Na^+^/K^+^ APTase involved in setting up Na^+^ and K^+^ electrochemical gradient, is enriched in the magenta cluster. DKK3, a head inducer and also a Wnt inhibitor, as well as a pro-apoptotic BEX2 gene are enriched in the magenta cluster, associated with neuronal maturation. Interestingly, genes involved in mitochondrial electron transport such as NDUFA8, NDUFC2, and UQCRB are also enriched in the magenta cluster, suggesting that alterations in mitochondrial electron transport machinery participate in neuronal maturation. As NDUFC2 and UQCRB appeared to be neuronal specific, this could underlie the fact that mature neurons consume large amount of energy, which was supplied by the extraordinary mitochondrial electron transport machinery that neurons specifically poses (Fig. 5).

### Identification of candidate genes that potentially mark the maturity of neurons

Through WGCNA, we have already identified a neuronal specific blue module (Fig. 2F–H). We overlapped genes in the magenta cluster and those in the blue module and found 138 neuronal specific genes that were affiliated with neuronal maturation. GO terms of this overlapping cluster are similar to that of the magenta cluster, such as “Neuron development” (CNTN1, FAIM2), “Cellular localization” (PDGFA, SEC62), “Action potential” (ATP1B1), and “Mitochondrial electron transport” (NDUFC2, UQCRB).

Since the 20 neurons we analyzed were all from cultured human neurons differentiated *in vitro* from more primitive stem cells, we wonder whether the neuronal maturation genes (138) we identified could be used generally to mark neuronal maturity. To explore this, we compared recently published data from Ben Barres’ laboratory by Darmanis et al. in *PNAS*, where human single neurons from fetal and adult human brains were compared. Darmanis reported 3 dimensional PCA of their data and revealed that dimension 1 could be used to demarcate mature (from adult brain) and immature (from fetal brain) neurons (Darmanis et al., 2015), but without electrophysiological recordings. When we evaluated expression of 1479 genes included in Dim1 by Darmanis et al. in our single neuron samples, we found a subset of the genes had enriched expression in our “matured” neuronal samples (Fig. S2). By cross-referencing data from Darmanis et al. and the 138 maturation genes identified in our present work, we isolated 39 genes shared by the two studies, which could potentially serve as generic biomarkers for human neuronal maturation. Among these 39 genes, “mitochondria electron transport” (NDUFC2 and UQCRB) stood out. Moreover, UCHL1, BEX2, PDGFA, FAIM2, CNTN1, SEC62, ATP1B1, and CALM2 are of particular interest, as they not only reappeared in the different rounds of aforementioned analyses but also had relatively high hub-ness in the network of the gene cluster.

## DISCUSSION

Coupled electrophysiological recording and transcriptome analyses on the same single neurons provided much more précised information on gene transcription programs of neurons with very specific electrophysiological functional properties. Such “patch-seq” technology effectively assisted identification of gene programs underlying neuronal maturation as judged by electrophysiological characteristics. It is comforting to discover that our cultured neurons derived from various human stem cell populations revealed a similar maturation program as that revealed by comparing human adult and fetal neurons in the brain, which indirectly reflected neuronal maturity. The 39 genes especially the 10 or so above highlighted genes may be particularly useful in the future to serve as generic biomarkers for evaluation of neuronal maturity.

We predict that coupled electrophysiological recordings and transcription analyses will be wildly used not only in human cell based “disease-in-dish” models to provide highly précised dissection of molecular mechanisms underlying the pathophysiology of diseased neurons with single cell resolution, but also in live brain slices and/or *in vivo* recording studies to pair electrophysiological properties with gene-expression analyses aiming to reveal the “molecular logic” of neural circuitry activities. However, it is possible that simple PCA-type of analyses will not reveal the underlying gene expression programs for specific physiological features one would like to study. In order to search for such programs, modified WCGNA methods as used in this particular study, will be extremely useful.

Along with the fast accumulation of brain mapping and connectome informations, anatomical zip-coding of the brain will soon become a reality. However, without knowing the molecular features of brain cells in each location, one would not easily detect physiological alterations or anything beyond anatomical changes in pathological conditions associated with the diseases, neither would one reveal molecular mechanisms underlying physiological changes related to learning, memory, all kinds of adaptive changes, as well as behavior, emotion, and thoughts. Coupling of brain mapping with single cell resolution transcriptome analyses will pave the way to our ultimate understanding of the brain and the mind.

## MATERIALS AND METHODS

### Neuron differentiation

hiPSCs and hESCs were differentiated into functional neurons following a modified version of published step-wise differentiation protocol(s). hESC- as well as hiPSC-derived neural stem/progenitor cell (NSC/NPC) colonies formed rosettes and expressed neural precursor markers Nestin and Sox2. Rapid conversion of hESC and hiPSC into functional induced neuronal (iN) cells could be achieved in less than two weeks by overexpression of exogenous Ngn2. The efficiencies of NPC differentiation into Tuj1-positive neurons were similar amongst all lines and were consistently close to 90% from culture to culture. Upon co-culture with mouse astrocytes, hESC/iPSC-derived neurons expressed mature neuronal markers, such as MAP2 as well as presynaptic Synapsin and vGlut1, with typical puncta patterns. Moreover, these neurons were able to produce action potentials, sodium and potassium currents upon stimulation. Spontaneous synaptic activity indicates that the neurons are relatively mature and form functional synaptic networks.

### Immunofluorescence and microscope analysis

Cells on coverslips were washed twice with PBS (5 min each), fixed with 4% PFA at room temperature (RT) for 10 min, then rewashed twice with PBS (5 min each). Coverslips were incubated for 1 h at RT in PBS, 0.03% Triton X-100, 3% BSA and 2% normal donkey serum (NDS, Jackson laboratory, #017-000-121) for permeablization and blocking non-specific antibody binding. Following that, incubation of primary antibodies was often carried out overnight at 4°C in the same buffer. Cells were then washed (3 times, 10 min each) in the same buffer and incubated with secondary antibodies (Jackson Laboratory) for 1 h at RT. Cells were finally washed with PBS (3 times, 10 min each) and mounted on glass slide. Staining was viewed and analyzed with Olympus up-right fluorescent microscope (BX51) or with confocal microscope (Leica TCS SP5 II**).** The following antibodies were used: mouse anti-Oct4 (Millipore), goat anti-Sox2 (Santa Cruz), mouse anti-Nestin (Chemicon), mouse anti-MAP2 (Sigma), rabbit anti-Synapsin (Synaptic Systems), mouse anti-vGlut1 (Synaptic Systems), Rabbit anti-GABA (Sigma).

### Morphological analyses

Recorded cells were filled with neurobiotin present in the intracellular solution via positive current injection (400 pA for 250 ms, 1.25 Hz) for at least 6 min. After recording, coverslips were fixed in 4% PFA 0.01 mol/L PBS for 10 min then rinsed with 0.01 mol/L PBS. Neurobiotin labeled cells were visualized via overnight incubation with 1:500 Cy3-conjugated streptavidin (Jackson Immuno Research Laboratories). Whole cell morphological reconstruction and analyses were carried out with Neurolucida software.

### Whole-cell recording

The coverslip plated with cultured cells was bathed in an RNase-free recording chamber filled with artificial cerebrospinal fluid (ACSF) containing (in mmol/L) 126 NaCl, 3 KCl, 1.2 NaH_2_PO_4_, 1.3 MgCl_2_, 2.4 CaCl_2_, 26 NaHCO_3_, and 10 glucose, bubbled with 95% O_2_/5% CO_2_. An upright infrared-differential interference contrast (IR-DIC) microscope (Olympus) equipped with epi-fluorescence illumination, an ORCA-R2 CCD camera, and two water immersion lenses (10× and 60×) were used to visualize and target recording electrode to cells. Glass recording electrodes (8–12 MΩ resistance) were filled with an intracellular solution consisting of (in mmol/L) 120 K-gluconate, 16 KCl, 2 MgCl_2_, 0.2 EGTA, 10 HEPES, 2.5 MgATP, 0.5 Na_3_GTP, and 10 Na-phosphocreatine (pH = 7.25 and 295 mOsm/kg). Recording was collected using MultiClamp 700B amplifier and pCLAMP10 software (Molecular Device), filtered at 2 KHz and sampled at 5 KHz. After establishing the whole-cell configuration, the spontaneous postsynaptic currents (sPSCs) were recorded for assessing the chemical connection between cells during voltage-clamp recording at −70 mV. To reveal intrinsic electrophysiological properties of the targeted cell, 500 ms current pulses (step at 2 pA) were injected into the cell to induce steady-state action potential (AP). Passive membrane properties were monitored periodically during the course of the experiments to ensure that the cell was healthy. Cells that showed significant rundown were discarded.

To catalog the intrinsic electrophysiological properties of cells, 6 parameters of AP were determined through Clampfit software (Molecular Device): (1) AP threshold determined from the membrane potential at the onset of AP; (2) rise time measured as the time between the threshold and the spike peak; (3) halfwidth determined as the time interval between its half-amplitude that reflects AP duration; (4) AP amplitude measured as the difference between the firing threshold to the peak; (5) firing rate determined as the maximal firing frequency of AP spikes; (6) input resistance (Rin) measured by dividing the initial averaged voltage deflection to the absolute value of first level of injected current pulse. The firing frequency and amplitude of sPSCs were analyzed using mini Analysis Program (Synaptosoft Inc.).

### Single Cell Harvest, cDNA and library preparation for next generation sequencing

After recording, the wider tip of another pipette filled with no more than 0.5 µL sterilized cell harvest solution containing (in mmol/L) 144 K-gluconate, 3 MgCl_2_, 0.5 EGTA and 10 HEPES (pH 7.20 and 295 mOsm/kg) was gently attached to the recorded cell, then a light suction (negative pressure) was applied, through a glass syringe (Thomas Scientific) connected to the pipette. The suction lasted until the entire cell entered the tip of the pipette. The moment the complete cell had been visually located inside the tip, the pipette was quickly removed from the bath. Content of the harvest pipette was expelled into a 0.2 mL PCR tube containing 4.5-µL pre-prepared lysis buffer. Preparation of single cell cDNAs was performed based on previously published protocols with modifications at several steps (Tang et al., 2009; Tang et al., 2010).

After the generation of cDNA from a single cell, 50 ng of cDNA was used for library preparation. Using the Covaris S2 System (Covaris), cDNA was sheared into about 125 bp short fragments according to the manufacturer’s instructions. Library preparation was using NEBNext Ultra DNA library Prep Kit for Illumina (E7370). After DNA fragmentation, end repair, adaptor ligation and PCR amplification for 8 cycles, libraries were used for Illumina deep sequencing. Reads were aligning to hg19 with TopHat2 (Kim et al., 2013). Gene expression levels were estimated by Cufflinks (Ghosh and Chan 2016). RNA-Seq data was deposited at GSE 77564.

### WGCNA

Weighted gene correlation network analysis (WGCNA) was performed as described before (Luo et al., 2015; Zhang et al., 2014). Briefly, a signed Pearson correlation network was constructed using any gene that was expressed at a FPKM value of 0.1 or higher in at least one of the samples. Soft power parameter was estimated and used to derive a pairwise distance matrix for selected genes using the topological overlap measure, and the dynamic hybrid cut method was used to detect clusters. The node centrality, defined as the sum of within-cluster connectivity measures, was used to rank genes for ‘‘hub-ness’’ within each cluster. For visual analysis of the constructed networks by hard thresholding of edge distances, usually the top 100 edges were represented using VisANT.

### Principle component analysis

PCA is performed with R package FactoMineR. Not detected (ND) values were replaced by the maximum value of the parameter when the parameter is negatively correlated with maturation, or replaced by the minimum value of the parameter when the parameter is positively correlated with maturation.

### Gene ontology term enrichment analysis

Fisher’s exact test was used to score GO terms for significant enrichment.

### Graphics

Unless otherwise specified, plots were generated in R.

## Electronic supplementary material

Supplementary material 1 (XLSX 12 kb)

Supplementary material 2 (PDF 288 kb)

Supplementary material 3 (AVI 861 kb)
