# Supplementary material for: Coupled electrophysiological recording and single cell transcriptome analyses revealed molecular mechanisms underlying neuronal maturation
Source: Protein Cell. 2016 Feb 16;7(3):175–86. doi: 10.1007/s13238-016-0247-8 (PMC4791425; doi:10.1007/s13238-016-0247-8)
Supplement: Supplementary file 2 — Supplementary material 2 (PDF 288 kb) [file 13238_2016_247_MOESM2_ESM.pdf]

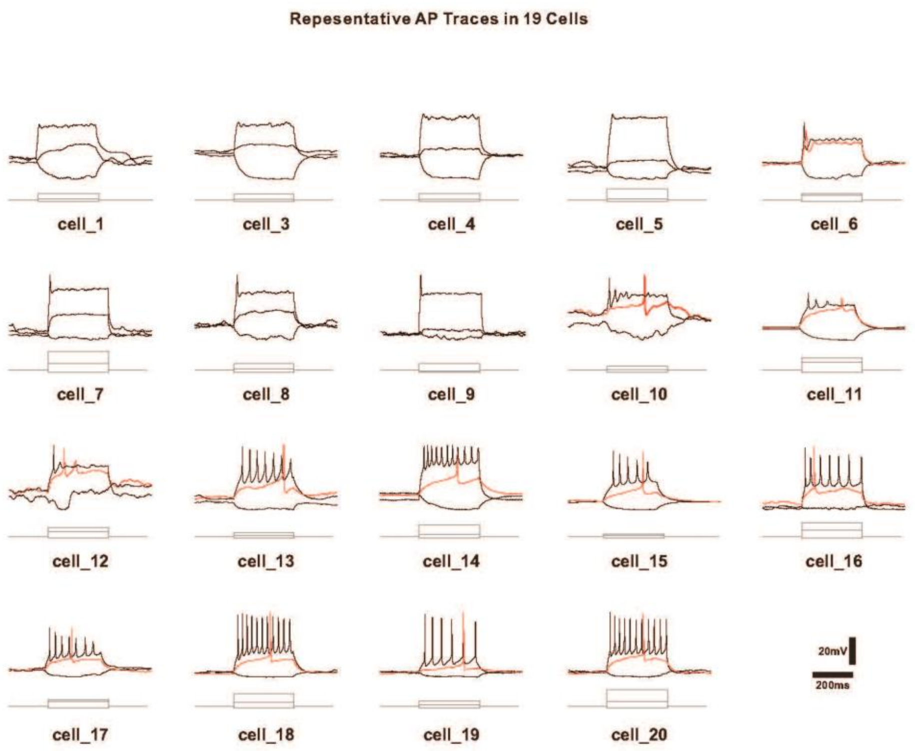

Supplementary Figure 1

Supplement Fig 1. Representative action potential responses of 19 studied human neurons evoked by current injection (Bars: 200 ms, 20 mv). The red trace represents the AP responses when the initial spike appeared during the injection.

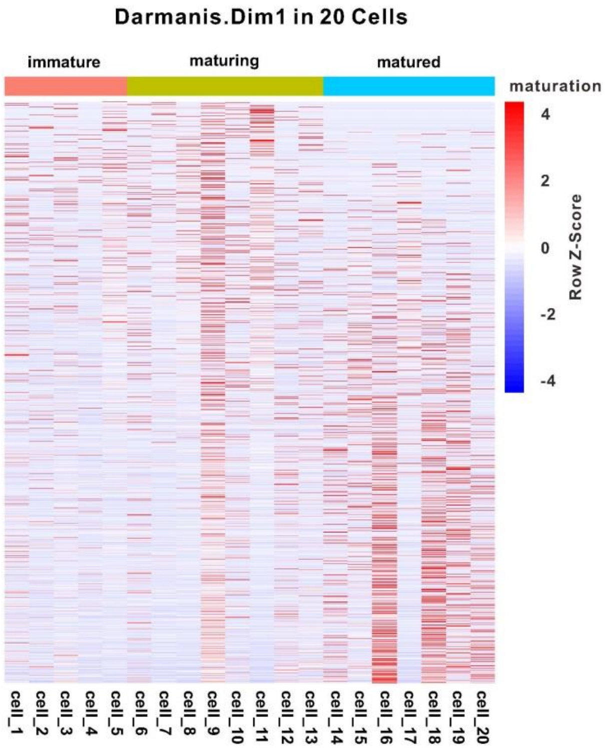

Supplementary Figure 2

Supplement Fig 2. Heatmap of the expresison of 1479 genes significantly associated with dimension 1 published by Darmanis et al in 20 studied cultured human neurons.

Supplement table 1. The 9 parameters of electrophysiological properties of human neurons
